# Residue Dissipation, Transformation Products, and Dietary Risk Assessment of Flonicamid in a Rice Paddy Ecosystem

**DOI:** 10.3390/foods15173104

**Published:** 2026-09-01

**Authors:** Yan Fu, Quansheng Wang, Liang Zhang, Yinliang Wu

**Affiliations:** 1 Ningbo Academy of Agricultural Sciences, Ningbo 315043, China; fuyan@zuaa.zju.edu.cn (Y.F.); wqsh1214@163.com (Q.W.); zl8755251@163.com (L.Z.); 2 Ningbo Key Laboratory of Testing and Control for Characteristic Agro-Product Quality and Safety, Ningbo 315043, China

**Keywords:** flonicamid, rice, residue dissipation, transformation products, dietary risk assessment

## Abstract

Flonicamid is a systemic pyridinecarboxamide insecticide used to control sap-sucking pests in agricultural crops. An ultra-high-performance liquid chromatography–tandem mass spectrometry (UHPLC–MS/MS) method was developed for the simultaneous determination of flonicamid and its metabolites, N-(4-trifluoromethylnicotinoyl)glycine (TFNG), 4-(trifluoromethyl)nicotinamide (TFNA-AM), and 4-(trifluoromethyl)nicotinic acid (TFNA) in rice plants, brown rice, rice husks, paddy soil, and paddy water. Method validation showed average recoveries of 73–115% with relative standard deviations of 1.1–9.9% across different matrices at three fortification levels. Field and laboratory experiments were conducted to characterize the dissipation, degradation, and transformation of flonicamid in the paddy ecosystem, and the dietary risk was assessed. Field dissipation followed first-order kinetics, with half-lives of 5.8, 2.9, and 7.1 days in rice plants, paddy soil, and paddy water, respectively. Under laboratory conditions, degradation proceeded markedly faster under aerobic than anaerobic conditions, with half-lives of 0.8–7.1 and 4.7–17.8 days in five typical Chinese soils, respectively. Six transformation products were identified by UHPLC–Q-TOF/MS coupled with UNIFI software (v1.9.4.0), including a novel product (M210) reported for the first time. At pre-harvest intervals of 7–21 days, terminal residues of flonicamid in brown rice were all below the Chinese maximum residue limit (MRL) of 0.1 mg/kg. Dietary risk assessment revealed that the acceptable daily intake percentages (%ADI) for 12 population subgroups ranged from 0.128% to 1.031% based on the parent compound, and from 0.404% to 3.808% under the EU residue definition. All values were far below 100%. These results indicate that flonicamid, when applied according to Good Agricultural Practice (GAP), poses a negligible dietary risk to consumers.

## 1. Introduction

Rice (*Oryza sativa* L.) is the staple food for more than half of the global population. Its production increasingly relies on intensive agrochemical inputs for pest and disease control, making pesticide residues a persistent threat to public health [1]. Neonicotinoid insecticides have been restricted in numerous countries owing to their adverse effects on non-target organisms such as bees [2], thereby driving the search for alternative insecticides. Flonicamid, a pyridinecarboxamide insecticide, has become an important option in integrated pest management because of its unique mode of action, high efficacy against piercing–sucking pests, and relative safety to natural enemies [3,4]. In China, flonicamid is currently registered on 28 crops, including rice, apple, tea, and cucumber, with more than 300 formulated products [5]. However, flonicamid is toxic to aquatic organisms, with reported effects including neurotoxicity [6,7]. It can also be transferred to fetuses via maternal–fetal transmission, with a detection rate of 59–87% in the first urine samples of newborns [8], and prenatal exposure has been associated with increased risks of childhood asthma, eczema, and neuroblastoma [9,10].

**Figure 1:**
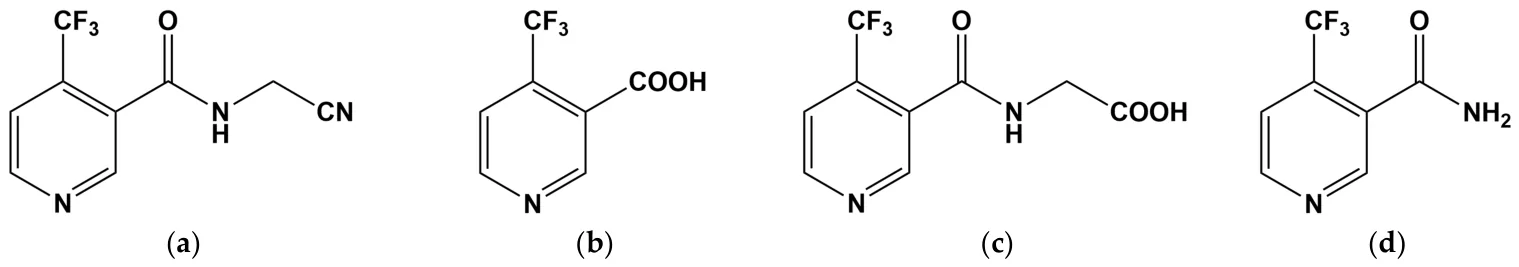
Chemical structures of flonicamid and its metabolites: (**a**) flonicamid; (**b**) 4-(trifluoromethyl)nicotinic acid (TFNA); (**c**) N-(4-trifluoromethylnicotinoyl)glycine (TFNG); (**d**) 4-(trifluoromethyl)nicotinamide (TFNA-AM).

The residue definition of flonicamid varies among countries and international organizations. For plant-derived commodities, China and the Joint FAO/WHO Meeting on Pesticide Residues (JMPR) define the residue as the parent compound alone [11], whereas the European Union and Japan define it as the sum of flonicamid, 4-(trifluoromethyl)nicotinic acid (TFNA), and N-(4-trifluoromethylnicotinoyl)glycine (TFNG), expressed as flonicamid. For products of animal origin, 4-(trifluoromethyl)nicotinamide (TFNA-AM) is additionally included in both the EU and Chinese definitions [12]. The chemical structures of flonicamid and its metabolites (TFNG, TFNA-AM, and TFNA) are shown in Figure 1. Flonicamid is a pro-insecticide: its insecticidal activity derives primarily from TFNA-AM, which can be more biologically active than the parent compound under certain conditions [13,14]. Monitoring the parent compound alone as the residue marker may therefore underestimate actual residue levels and, consequently, dietary exposure.

Analytical methods reported for flonicamid include gas chromatography (GC) [15], gas chromatography–mass spectrometry (GC–MS) [16], ultra-high-performance liquid chromatography–tandem mass spectrometry (UHPLC–MS/MS) [17,18,19,20,21,22], ultra-high-performance liquid chromatography–high-resolution mass spectrometry [23], and enzyme-linked immunosorbent assay (ELISA) [24]. UHPLC-MS/MS has emerged as a powerful analytical tool for the determination of pesticide residues in complex matrices [25]. The dissipation behavior and dietary risk have been studied in rice, cucumber, apple, peach, cabbage, and cotton [26,27,28,29,30]; however, methods for the simultaneous determination of flonicamid and its metabolites in agricultural products remain limited [31]. Banshtu et al. [32], Tian et al. [33], Qiao et al. [34], and Wang et al. [35] investigated the residue dissipation and dietary risk of flonicamid and its metabolites in okra, apple, kiwifruit, and cabbage, respectively, and found no appreciable health risk to consumers under supervised field conditions following good agricultural practice. Tan et al. [36] examined the residue distribution, influencing factors, and ecological risk of flonicamid and three metabolites in plants, soil, water, and sediment of a tropical rice–vegetable rotation system in Hainan, China. They identified the highest risks in soils, during vegetable planting periods, and in a central intensively planted area. Nevertheless, the dissipation dynamics, terminal residues, and dietary risk of flonicamid and its metabolites following application in the paddy ecosystem remain poorly understood.

In this study, a UHPLC–MS/MS method was therefore established for the simultaneous determination of flonicamid and its three metabolites in rice plants, brown rice, rice husks, soil, and water. Field trials were conducted to investigate the dissipation dynamics and terminal residues of a 20% flonicamid suspension concentrate, and the dietary risk was assessed based on Chinese dietary consumption data. In parallel, laboratory simulation experiments were performed to examine the degradation behavior of flonicamid under different conditions, and the transformation products were identified by ultra-high-performance liquid chromatography–quadrupole time-of-flight mass spectrometry (UHPLC–Q-TOF/MS). This study provides fundamental data to support the safe use and risk assessment of flonicamid in rice.

## 2. Materials and Methods

### 2.1. Reagents and Materials

Flonicamid standard (CAS 158062-67-0, 98.8%) was purchased from Dr. Ehrenstorfer GmbH (Augsburg, Germany). The metabolite standards TFNG (CAS 207502-65-6, 99.9%), TFNA-AM (CAS 158062-71-6, 97.9%), and TFNA (CAS 158063-66-2, 94.9%) were purchased from Hayashi Pure Chemical Ind., Ltd. (Osaka, Japan). Leucine enkephalin was obtained from Waters (Milford, MA, USA). Acetonitrile (HPLC grade) was purchased from Merck (Darmstadt, Germany), formic acid (analytical grade) from Aladdin (Shanghai, China), and acetic acid (analytical grade) from Sinopharm Chemical Reagent Co., Ltd. (Shanghai, China). Ultrapure water was prepared using a Milli-Q system (Millipore, Bedford, MA, USA).

Analyses were performed on a Xevo TQ-S ultra-high-performance liquid chromatography–tandem quadrupole mass spectrometer and a Xevo G2-S Q-TOF ultra-high-performance liquid chromatography–quadrupole time-of-flight mass spectrometer (both from Waters, Milford, MA, USA). Other equipment included a KS 4000 ic temperature-controlled shaker and a Genius 3 vortex mixer (IKA, Staufen, Germany), a Sigma 3K15 high-speed centrifuge (Sigma, Osterode, Germany), and an XPE205 analytical balance (Mettler-Toledo, Greifensee, Switzerland).

### 2.2. Field Trial

The field trial was conducted from May to July 2024 in Ningbo, Zhejiang Province, China (29°48′58″ N, 120°38′34″ E), using the rice cultivar Yongxian 15. The site has a subtropical monsoon climate with four distinct seasons, an annual mean temperature of 16.4 °C, annual precipitation of 1480 mm, and annual sunshine duration of 1850 h. The soil at the experimental site was a paddy soil with a pH of 5.4, an organic matter content of 6.32 g/kg, and a cation exchange capacity (CEC) of 12.0 cmol/kg. According to Good Agricultural Practices (GAP), the recommended application rate of the 20% flonicamid suspension concentrate (SC) for rice planthopper control is 60–75 g a.i./ha, applied once by foliar spraying. Rice growth stages at application were coded according to the Biologische Bundesanstalt, Bundessortenamt und CHemische Industrie (BBCH) scale [37], and meteorological conditions (air temperature, relative humidity, wind speed, and rainfall before and after each application) were recorded [38]. The details for each application event are summarized in Supplementary Table S1.

For the dissipation study, plots of 100 m^2^ were sprayed uniformly with flonicamid at 112.5 g a.i./ha (1.5 times the maximum recommended dose) at the stem elongation stage (BBCH 31), with three independent replicate plots. Flonicamid was applied using a commercially available electric knapsack sprayer (Model 3WBD-16, Lanyi, Xuzhou, China) equipped with a hollow cone nozzle. The spray solution was prepared to target a theoretical application volume of 750 L/ha (75 mL/m^2^). Prior to application, the sprayer flow rate was verified by collecting spray liquid for 20 s in triplicate; the average flow rate ranged from 21.10 to 21.87 mL/s, with a maximum deviation below 2.89%. The applicator’s walking speed was calibrated before application to ensure that the actual spray volume corresponded to the target theoretical volume. A continuous shallow water layer of approximately 3 cm was maintained in the paddy plots throughout the dissipation experiment. Rice plants were sampled at 2 h and 1, 3, 5, 7, 14, 21, and 28 days after application; paddy soil at 2 h and 1, 3, 5, 7, 14, and 21 days; and paddy water at 2 h, 8 h, and 1, 2, 3, 5, 7, and 14 days.

The dissipation and terminal residue trials were conducted in separate plots. For the terminal residue trial, flonicamid was applied once at 75 g a.i./ha (the maximum recommended dose) or 112.5 g a.i./ha (1.5 times the recommended dose) during the dough stages (BBCH 83–87). Rice straw and rice ears (subsequently separated into brown rice and husks) were collected at pre-harvest intervals (PHIs) of 7, 14, and 21 days after application. An alternate wetting and drying irrigation regime was adopted in this trial, and field water was completely drained 7 days before harvest to facilitate grain ripening. Each treatment was performed in three independent field plots separated by 3-m-wide buffer zones, and an untreated control was included.

All samples were collected from 12 random sampling points within each plot. Rice plant and straw samples (0.5 kg each) were cut into pieces smaller than 1 cm and reduced to 100 g by quartering. Paddy soil samples (1 kg each) were collected from the 0–10 cm layer, cleared of plant residues and gravel, homogenized, and reduced to 200 g by quartering. Paddy water samples (2 L in total per plot) were mixed thoroughly, and a 500-mL subsample was taken. Rice ears (1 kg per sample) were dehulled, and the resulting brown rice and husks were separately homogenized and reduced to 100 g each by quartering. All samples were transported to the laboratory within 8 h of collection and stored at −20 °C or below until analysis.

### 2.3. Laboratory Degradation Experiments

#### 2.3.1. Soil Degradation Experiment

Five typical soils were collected from the 0–10 cm surface layer of agricultural fields in Hunan, Jilin, Zhejiang, and Hebei provinces of China. These soils had no history of flonicamid application. The soil samples were stored in the dark after air drying, homogenization, and sieving (2 mm), followed by a 14-day pre-incubation period [39,40]. Their physicochemical properties are listed in Table 1. The fortified concentration of 5 mg/kg was selected to achieve reliable analyte quantification and valid kinetic fitting, representing a worst-case exposure scenario according to GB/T 31270.1-2014 (operative at the time of experimentation). The fortified soils were thoroughly blended and equilibrated before initial sampling to ensure homogeneous distribution of flonicamid. Degradation was studied under both aerobic and anaerobic conditions. For aerobic incubation, ultrapure water was added to maintain soil moisture at 60% of maximum water-holding capacity, with vessels covered with breathable film for gas exchange. For anaerobic incubation, additional sterile ultrapure water was added as an overlay to form an approximately 2-cm standing water layer above the soil, and these vessels were tightly sealed with rubber stoppers. All treatments were incubated at 25 ± 1 °C in the dark. At 0, 1, 3, 5, 7, 14, 21, 28, and 35 days after treatment, three replicate incubation vessels were sampled; the soil samples were then freeze-dried and stored at −20 °C until analysis.

#### 2.3.2. Hydrolysis Experiment

A stock solution of flonicamid in acetonitrile was diluted with sterile ultrapure water or sterile buffer solutions (pH 4, 7, and 9) to obtain four test solutions at 1 mg/L. After thorough vortexing, the solutions were transferred in sealed amber bottles and incubated in the dark at 25 or 50 °C. Each treatment was prepared in three independent replicates. Samples were collected at 0, 1, 3, 5, 7, 14, 21, 28, 35, 42, and 60 days to determine the residual concentration of flonicamid.

### 2.4. Sample Preparation Procedure

Brown rice and soil samples (5.0 g), or rice husk and straw samples (2.5 g), were weighed into 50 mL centrifuge tubes. After adding 10 mL of ultrapure water, the tubes were vortexed and allowed to stand for 15 min. Subsequently, 20.0 mL of acetonitrile containing 2.5% acetic acid was added, and the tubes were shaken at 350 rpm for 30 min, followed by centrifugation at 9500 rpm for 3 min. For water samples, 10 mL of water or buffer and 20 mL of acetonitrile containing 0.5% formic acid were added to a centrifuge tube. The mixture was then shaken and centrifuged as described above. An aliquot (0.2 mL) of the supernatant was diluted to 1 mL with 0.1% formic acid, filtered through a 0.22 μm nylon membrane (Dikma Technologies, Beijing, China), and analyzed by LC-MS/MS or UHPLC-Q-TOF/MS.

### 2.5. Instrumental Conditions

#### 2.5.1. LC–MS/MS Conditions

Chromatographic separation was achieved on an Acquity UPLC BEH C18 column (2.1 mm × 100 mm, 1.7 µm; Waters, Milford, MA, USA) maintained at 35 °C, with the sample compartment held at 15 °C. Gradient elution was carried out using 0.1% formic acid in water (mobile phase A) and acetonitrile (mobile phase B) as follows: 80% A at 0–0.5 min, decreased to 20% A at 1.0 min and held for 2 min, then returned to 80% A at 3.1 min and re-equilibrated for 1.9 min (total run time 5.0 min). The flow rate was set at 0.3 mL/min. The injection volume was 10 µL.

Mass spectrometric analysis was performed on a triple quadrupole mass spectrometer equipped with an electrospray ionization (ESI) source operated in positive ion mode. The capillary voltage was set at 2.5 kV. The source and desolvation temperatures were maintained at 150 °C and 500 °C, respectively. The desolvation gas flow rate was 800 L/h, and the cone gas flow rate was 50 L/h. Analytes were monitored in multiple reaction monitoring (MRM) mode, and the specific MRM parameters are listed in Table 2.

#### 2.5.2. High-Resolution Mass Spectrometry Conditions

High-resolution mass spectra were acquired using an electrospray ionization (ESI) source operated in positive ion and resolution mode over a mass range of *m*/*z* 50–1200. Mass calibration was performed with leucine enkephalin (*m*/*z* 556.2771 in positive ion mode) as the lock mass, with a scan time of 0.25 s. The instrumental parameters were set as follows: cone voltage, 40 V; capillary voltage, 1.0 kV; source temperature, 150 °C; desolvation temperature, 350 °C. Nitrogen was used as both the drying and nebulizing gas, with cone and desolvation gas flows of 50 and 800 L/h, respectively.

### 2.6. Calibration and Method Validation

Stock solutions (1000 mg/L) of flonicamid, TFNG, TFNA-AM, and TFNA were individually prepared by dissolving each compound in acetonitrile. All stock solutions were stored at −18 °C or below in the dark and used within one year. A mixed intermediate standard solution (100 mg/L) was then prepared by combining 1.0 mL of each stock solution and diluting the mixture to 10 mL with acetonitrile. This solution was stored at 4 °C and used within three months. Matrix-matched standard working solutions at 0.0001, 0.0005, 0.001, 0.005, 0.01, and 0.05 mg/L were freshly prepared for brown rice, rice husk, rice plant, soil, and water by diluting the mixed intermediate standard solution with the corresponding blank extracts. Calibration curves were generated by plotting peak area against concentration (mg/L) to evaluate the linear relationship.

The method was validated for accuracy and precision in accordance with NY/T 788—2018. Blank brown rice, rice husk, rice plant, and soil samples were spiked with mixed standard solutions of flonicamid and its metabolites at 0.01, 0.1, and 5 mg/kg; water samples were spiked at 0.005, 0.1, and 1 mg/L. Five replicates were prepared for each matrix at each level and analyzed as described above.

### 2.7. Data Analysis

Data acquisition and processing were performed using MassLynx software (v4.1). Dissipation of flonicamid under different environmental conditions was described by a first-order kinetic model, and curve fitting and data organization were carried out in WPS Office. The first-order kinetic equation and the half-life were calculated as follows [41]:

C*_t_* = C_0_e^−*kt*^ (1)

*t*_0.5_ = ln2/*k* (2)

where C_0_, C*_t_* are the initial concentration and the concentration at time *t*, respectively; *k* is the dissipation rate constant; and *t*_0.5_ is the half-life.

## 3. Results

### 3.1. Analysis Method Validation

**Figure 2:**
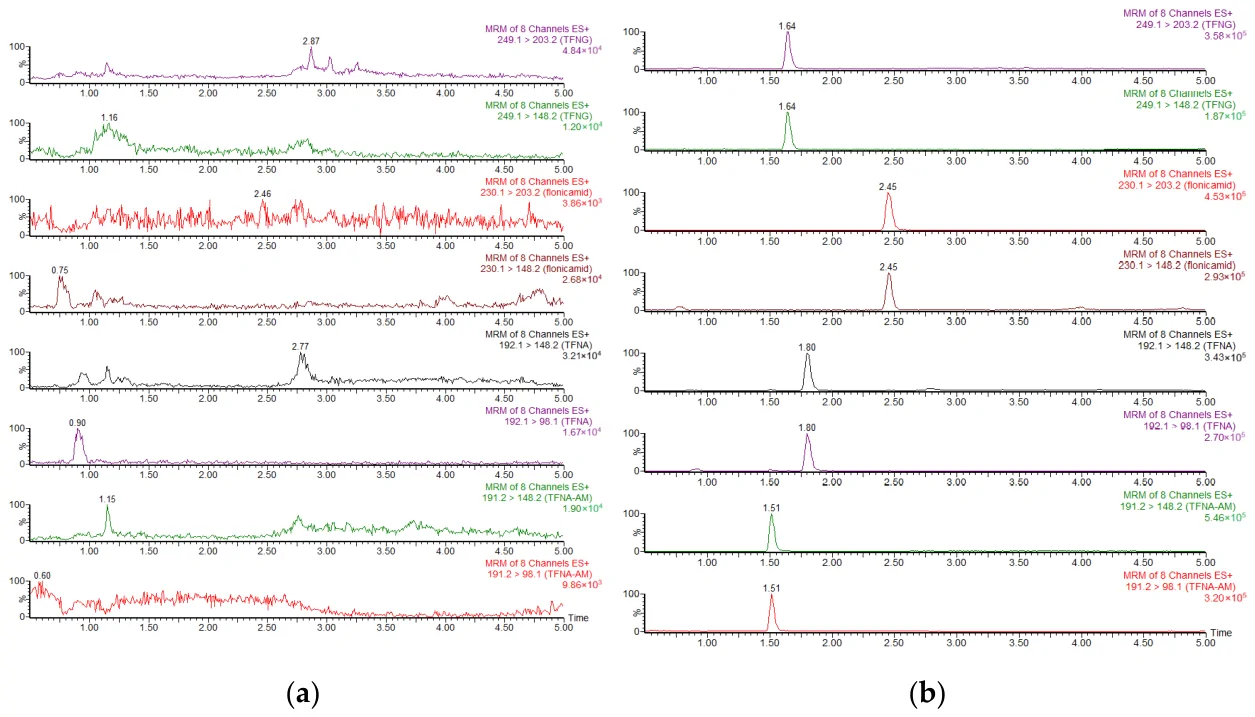
Representative MRM chromatograms of (**a**) blank brown rice and (**b**) matrix-matched standard of flonicamid and its metabolites at 0.0005 mg/L.

Linearity was assessed using matrix-matched calibration curves over the concentration range of 0.0001–0.05 mg/L for flonicamid and its three metabolites (TFNG, TFNA-AM, and TFNA). All calibration curves showed good linearity with coefficients of determination (*R*^2^) ≥ 0.999 (Table S2), and the corresponding matrix-matched curves are provided in Figure S1. Method accuracy and precision were evaluated through recovery experiments on rice plant, rice husk, brown rice, water, and soil at three spiking levels, each with five replicates. Mean recoveries for brown rice, rice husk, and rice plant ranged from 73% to 115%, with relative standard deviations (RSDs) of 1.1–9.9%; those for water and soil ranged from 73% to 108%, with RSDs of 1.3–9.2% (Table 3). Representative MRM chromatograms are presented in Figure 2. These results demonstrate that the method is suitable for the simultaneous determination of flonicamid and its three metabolites in rice and environmental matrices, with accuracy and precision meeting the requirements of the guideline on pesticide residue trials (NY/T 788-2018).

### 3.2. Dissipation Dynamics of Flonicamid and Its Metabolites in the Paddy Ecosystem

**Figure 3:**
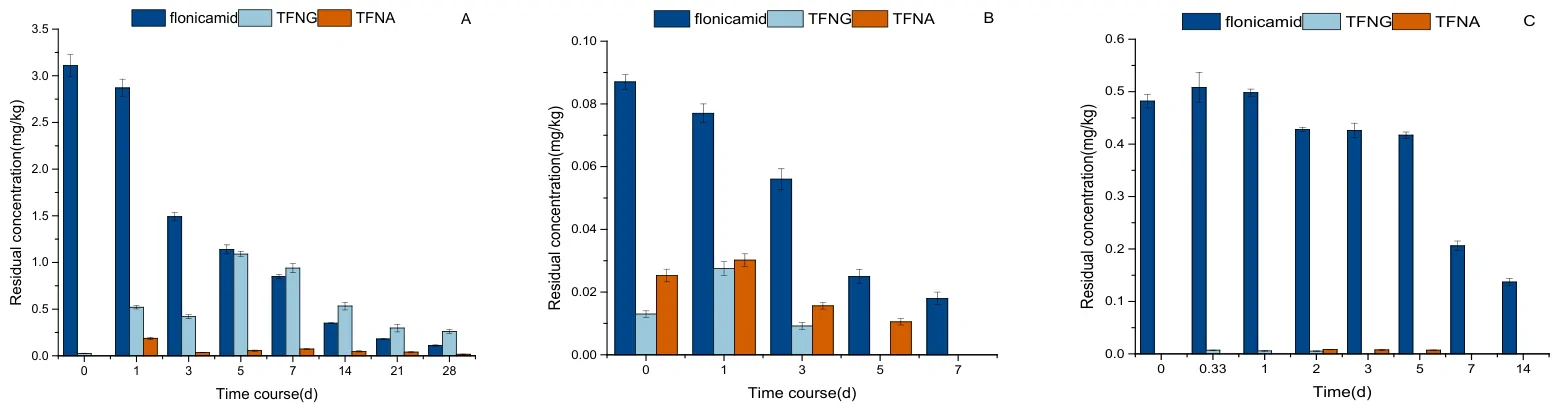
Residues of flonicamid and its metabolites in rice plants (**A**), paddy soil (**B**), and paddy water (**C**).

To clarify the dissipation behavior of flonicamid and its metabolites in the paddy ecosystem, their distribution and dissipation kinetics were investigated in a field trial. The initial distribution of flonicamid followed the order rice plant > paddy water > paddy soil (Figure 3), with initial concentrations of 3.11 mg/kg in rice plants, 0.482 mg/kg in paddy water, and 0.087 mg/kg in paddy soil. Dissipation of flonicamid in all three matrices followed first-order kinetics (Figure 3, Table 4), with half-lives of 5.8, 7.1, and 2.9 days in rice plants, paddy water, and paddy soil, respectively.

In rice plants (Figure 3A), flonicamid dissipated rapidly, with dissipation rates exceeding 50% within 3 days and reaching 71.7% at 7 days. The half-life in rice plants (5.8 d) was considerably longer than that reported by Chauhan et al. [26] for rice in Gujarat, India (0.49–0.52 d), which may be attributed to differences in climatic conditions, rice varieties, and cultivation practices. The metabolite TFNA was first detected on day 1 and reached a maximum of 0.073 mg/kg on day 7, while TFNG exhibited a similar pattern of initial increase followed by decline, peaking at 1.09 mg/kg on day 5. Thus, TFNG was the predominant primary metabolite in rice plants, whereas TFNA-AM was not detected throughout the sampling period. This is consistent with findings in bell pepper, apple, and cabbage [23,33,35], suggesting that TFNA-AM is unstable under field conditions or its concentration remains below the method detection limit.

In paddy soil (Figure 3B), flonicamid dissipated most rapidly, with a dissipation rate of 79.3% at 7 days, and both TFNG and TFNA were detected on the day of application. The dissipation in soil was markedly faster than that reported by Liu et al. [27] in cucumber and apple soils (10.3–14.2 d), which may be associated with differences in soil type, organic matter content, pH, microbial community structure, and temperature and moisture conditions [27,35].

In paddy water (Figure 3C), flonicamid dissipation was relatively slow, reaching only 13.5% at 5 days, with trace amounts of TFNG and TFNA detected. In summary, the dissipation behavior of flonicamid in the paddy ecosystem exhibited matrix-dependent differences, with the overall dissipation rate following the order paddy soil > rice plants > paddy water.

### 3.3. Degradation Kinetics of Flonicamid in Water and Soil Under Laboratory Conditions

Laboratory simulation experiments were conducted to investigate the degradation behavior of flonicamid in four aqueous solutions and five typical Chinese soils. Flonicamid was essentially stable in ultrapure water, pH 4 and pH 7 buffer solutions, whereas its hydrolysis rate increased with increasing alkalinity and temperature, which is consistent with the hydrolysis behavior reported by Lü et al. [42]. The degradation of flonicamid in the pH 9 buffer solution and in the five soils followed first-order kinetics, and the kinetic parameters and half-lives are summarized in Table 5. Under aerobic conditions, the degradation half-lives followed the order fluvo-aquic soil (0.8 d) < black soil (1.9 d) < cinnamon soil (2.5 d) < red soil (5.1 d) < paddy soil (7.1 d). Under anaerobic conditions, the corresponding half-lives were considerably longer, following the order fluvo-aquic soil (4.7 d) < black soil (11.2 d) < cinnamon soil (11.6 d) < red soil (13.3 d) < paddy soil (17.8 d). Thus, degradation proceeded markedly faster under aerobic than anaerobic conditions in all five soils. Soil pH and organic matter content appeared to be the key factors governing flonicamid degradation. Flonicamid degraded most rapidly in fluvo-aquic soil, which had the highest pH (8.46) among the five soils despite a moderate organic matter content (13.2 g/kg). The second-fastest degradation occurred in black soil, which had the highest organic matter content (33.6 g/kg). These observations suggest that, for the soils tested, the promoting effect of alkaline conditions on flonicamid degradation may outweigh that of organic matter. This is supported by López-Ruiz et al. [43], who demonstrated that flonicamid is chemically stable under acidic conditions but is rapidly converted to TFNG via a N-(4-Trifluoromethylnicotinoyl)glycinamide (TFNG-AM) intermediate under alkaline conditions.

Transformation products were identified by UHPLC-Q-TOF/MS, and their structures were proposed using UNIFI software (v1.9.4.0). The confidence of each structural assignment was categorized following the scheme of Schymanski et al. [44]: level 1, structure unambiguously confirmed against an authentic reference standard; level 2b, probable structure inferred from diagnostic fragmentation evidence; and level 3, one or more tentative candidate structures. Six transformation products were detected in the water and soil samples, of which five have been previously reported and one (M210) was identified for the first time in this study (Table 6). M210 exhibited a molecular ion at *m*/*z* 211 with characteristic fragment ions at *m*/*z* 183 and 128, and was tentatively assigned as 5,5-difluoro-5H-pyrido [3,4-c]azepine-6,9-dione based on its fragmentation pattern. The MS spectrum and proposed structures of the major fragments of M210 are shown in Figure S2. The degradation of flonicamid primarily involved amide hydrolysis, nitrile hydrolysis, defluorination, and hydroxylation.

### 3.4. Terminal Residues of Flonicamid and Its Metabolites

Terminal residues under the two application doses are summarized in Table 7. In rice straw, at 75 g a.i./ha with a single application, the average residues of the parent flonicamid at 7, 14, and 21 days after application ranged from 0.027 to 0.27 mg/kg, those of TFNG from 2.2 to 2.9 mg/kg, and those of TFNA from 0.040 to 0.060 mg/kg. At 112.5 g a.i./ha, the corresponding ranges were 0.13–0.63 mg/kg for flonicamid, 4.2–6.4 mg/kg for TFNG, and 0.10–0.31 mg/kg for TFNA. Over the 7–21 day period, residues of the parent flonicamid and TFNG in straw declined gradually, whereas TFNA tended to increase, and TFNA-AM remained undetected (<0.01 mg/kg).

In rice husks, at 75 g a.i./ha, the average residues of the parent flonicamid, TFNG, TFNA, and TFNA-AM ranged from 1.2 to 1.7, 0.30 to 0.63, 0.050 to 0.070, and 0.012 to 0.037 mg/kg, respectively. At 112.5 g a.i./ha, the corresponding ranges were 1.9–2.7, 0.80–1.0, 0.11–0.14, and 0.025–0.037 mg/kg, respectively. Over the 7–21 day period, the parent flonicamid residues in rice husks decreased with time, while TFNG, TFNA, and TFNA-AM were consistently detected. The residue levels followed the order flonicamid > TFNG > TFNA > TFNA-AM.

In brown rice, at 75 g a.i./ha, the average residues of the parent flonicamid and TFNG ranged from 0.024 to 0.035 mg/kg and from 0.043 to 0.15 mg/kg, respectively. At 112.5 g a.i./ha, flonicamid residues ranged from 0.049 to 0.072 mg/kg and TFNG from 0.090 to 0.19 mg/kg. TFNA was not detected at 7 days but was detected at 14 and 21 days, with average residues of 0.016 and 0.021 mg/kg, respectively. TFNA-AM was not detected in brown rice. Notably, TFNG residues in brown rice increased over the 7–21 day period, whereas parent flonicamid residues remained at generally low levels.

The residue distribution of flonicamid and its metabolites differed markedly among the rice fractions. In straw, TFNG was the dominant residue, present at levels substantially higher than the parent compound. In rice husks, the parent flonicamid accounted for the highest residues. TFNA-AM was detected only in rice husks, at low levels, but was not found in straw or brown rice, suggesting that this metabolite is mainly distributed in the outer protective tissues of the grain. In all three fractions, residues increased with application dose, showing a clear positive dose–residue relationship. China currently defines the residue definition of flonicamid for monitoring purposes as the parent compound, with a maximum residue limit (MRL) of 0.1 mg/kg in brown rice [45]. Under the application regime used in this trial, the terminal residues of flonicamid in brown rice did not exceed the MRL.

### 3.5. Dietary Risk Assessment

The dietary intake risk of flonicamid and its metabolites was assessed based on the terminal residues in brown rice (Table 7) and the daily rice consumption of Chinese residents from the WHO Global Environment Monitoring System–Food Contamination Monitoring and Assessment Programme (GEMS/Food). The Joint FAO/WHO Meeting on Pesticide Residues (JMPR) has established an acceptable daily intake (ADI) of 0.07 mg/kg bw for flonicamid but no acute reference dose (ARfD) [11]; therefore, only the long-term dietary intake risk was evaluated. The risk was expressed as the national estimated daily intake (NEDI) relative to the ADI (%ADI) [20]. Because the food-consumption-to-body-weight ratio differs among population groups, dietary exposure and the associated risk vary considerably. Twelve population subgroups were therefore assessed, comprising males and females in six age groups (0–35 months, 3–5, 6–14, 15–49, 50–74, and >75 years). Residue definitions also differ among jurisdictions. The European Union and Japan define flonicamid residues as the sum of flonicamid, TFNA, and TFNG, expressed as flonicamid, whereas the Chinese registration residue definition and the JMPR report define the residue in plant-derived commodities as the parent compound only. Risk was therefore assessed under both definitions.

For brown rice harvested at pre-harvest intervals (PHIs) of 7, 14, and 21 days, the %ADI values of the 12 subgroups ranged from 0.128% to 1.031% when only the parent compound was considered (Table 8), well below the 100% threshold. When TFNA and TFNG were included according to the EU/Japanese definition, the %ADI for the sum of the three compounds ranged from 0.404% to 3.808%. These values were 2.3–6.9 times higher than those based on the parent compound alone, but remained far below 100%. Across both definitions and all PHIs, the highest %ADI values were consistently observed in infants aged 0–35 months, reaching 1.031% for the parent compound (PHI 14 d, high dose) and 3.808% for the sum of the three compounds (PHI 21 d, high dose). These results indicate that the application of flonicamid according to Good Agricultural Practice (GAP) for rice pest control poses no unacceptable long-term dietary risk to any population subgroup, including infants and young children. This conclusion agrees with previous findings on cabbage, cucumber, and apple, where dietary risks of flonicamid were also acceptable [27,35]. It should be noted, however, that this assessment considered rice consumption only, and the cumulative exposure from all registered crops warrants further evaluation.

Limitations of this study should be noted. First, all field trials were conducted at a single site within one growing season; multi-site and multi-season studies are therefore needed to improve the generalizability and robustness of the results. Second, laboratory microcosms cannot fully reproduce the complexity of natural paddy soils, including rhizosphere effects and fluctuating redox conditions. Finally, metabolite M210 was only tentatively identified by TOF-MS, and further structural confirmation is warranted in future studies.

## 4. Conclusions

A multi-residue LC-MS/MS method was developed for the simultaneous determination of flonicamid and its three metabolites (TFNG, TFNA-AM, and TFNA) in rice plant, brown rice, rice husk, soil, and water. The accuracy and precision of the method met the requirements of the guidelines for pesticide residue analysis. Field dissipation trials showed that flonicamid dissipated with half-lives of 5.8, 2.9, and 7.1 days in rice plants, paddy soil, and paddy water, respectively. Under laboratory conditions, the half-lives in five typical Chinese soils ranged from 0.8 to 7.1 days and from 4.7 to 17.8 days under aerobic and anaerobic conditions, respectively. Flonicamid was essentially stable in acidic and neutral buffer solutions, while hydrolysis was accelerated under alkaline conditions. Six transformation products were identified by UHPLC-Q-TOF/MS, including a novel product tentatively assigned as 5,5-difluoro-5H-pyrido [3,4-c]azepine-6,9-dione (M210). At harvest, residues in brown rice (0.024–0.072 mg/kg) were below the Chinese maximum residue limit of 0.1 mg/kg. The %ADI values for the 12 population subgroups ranged from 0.128% to 1.031% based on the parent compound, and from 0.404% to 3.808% under the EU residue definition—all well below 100%. These results indicate that flonicamid, when applied according to GAP, dissipates readily in the paddy ecosystem and poses a negligible dietary risk to Chinese consumers.

## Figures and Tables

**Table 1:** Physicochemical properties of the tested soils.

Soil Type	Location	pH	Organic Matter/(g/kg)	Soil Texture	CEC/(cmol/kg)
Red soil	Hunan	4.6	3.45	clay	15.2
Black soil	Jilin	7.96	33.6	silty clay	27.8
Paddy soil	Zhejiang	4.7	28.1	silty clay loam	13.0
Fluvo-aquic soil	Langfang, Hebei	8.46	13.2	clay loam	16.9
Cinnamon soil	Tangshan, Hebei	6.66	8.96	clay loam	13.4

**Table 2:** UHPLC–MS/MS parameters.

Compound	Precursor Ion (*m*/*z*)	Product Ion (*m*/*z*)	Dwell Time (s)	Cone Voltage (V)	Collision Energy (eV)
Flonicamid	230.1	148.2	0.05	30	24
		203.2 *	0.05	30	15
TFNG	249.1	148.2	0.05	30	27
		203.2 *	0.05	30	15
TFNA-AM	191.2	98.1	0.05	30	27
		148.2 *	0.05	30	23
TFNA	192.1	98.1	0.05	30	25
		148.2 *	0.05	30	22

* Ions are used for quantification.

**Table 3:** Average recoveries and RSDs of flonicamid and its metabolites in different matrices (n = 5).

Matrix	Spiked Concentration /(mg/kg)	Flonicamid		TFNG		TFNA-AM		TFNA	
		Average Recovery /%	RSD /%	Average Recovery /%	RSD /%	Average recovery /%	RSD /%	Average Recovery /%	RSD /%
Brown rice	0.01	96	2.5	113	2.7	89	2.1	85	1.9
	0.1	100	1.1	103	1.6	98	1.4	87	1.9
	5	105	2.1	98	3.4	95	3.6	89	1.5
Rice husk	0.01	109	2.5	106	1.7	108	6.1	79	9.9
	0.1	115	2.5	112	2.2	97	6.8	87	5.7
	5	85	2.9	101	3.1	90	3.0	73	5.8
Rice plant	0.01	78	5.3	111	3.1	95	4.2	105	2.1
	0.1	87	8.1	112	5.1	109	4.2	105	2.5
	5	102	4.2	82	4.7	96	5.1	91	7.9
Soil	0.01	105	2.1	92	9.2	74	5.4	73	8.1
	0.1	103	2.5	99	1.5	93	4.8	87	4.3
	5	91	7.1	92	7.6	99	5.4	93	4.9
water	0.005	102	4.1	105	3.3	97	3.3	99	4.8
	0.1	96	1.3	101	4.9	95	4.8	98	2.6
	1	100	3.9	108	7.2	101	5.0	100	3.7

**Table 4:** Dissipation kinetics of flonicamid in the paddy ecosystem.

Matrix	Dissipation Equation	Coefficient of Determination (*R*^2^)	Half-Life (d)
Rice plant	C*_t_* = 2.4151e^−0.120*t*^	0.965	5.8
Paddy soil	C*_t_* = 0.0953e^−0.240*t*^	0.967	2.9
Paddy water	C*_t_* = 0.5310e^−0.098*t*^	0.912	7.1

**Table 5:** Degradation kinetic parameters of flonicamid in pH9 buffer solution and five soils.

Sample	Treatment	Degradation Equation	Coefficient of Determination (*R*^2^)	Half-Life (d)
pH9 solution	25 °C	C*_t_* = 1.0179e^−0.003^*^t^*	0.931	231
	50 °C	C*_t_* = 1.0635e^−0.064^*^t^*	0.966	10.8
Red soil	aerobic	C*_t_* = 3.8622e^−0.135*t*^	0.965	5.1
	anaerobic	C*_t_* = 4.4690e^−0.052*t*^	0.978	13.3
Black soil	aerobic	C*_t_* = 3.7917e^−0.363*t*^	0.959	1.9
	anaerobic	C*_t_* = 4.6670e^−0.062*t*^	0.948	11.2
Paddy soil	aerobic	C*_t_* = 3.8953e^−0.098*t*^	0.939	7.1
	anaerobic	C*_t_* = 4.1468e^−0.039*t*^	0.959	17.8
Fluvo-aquic soil	aerobic	C*_t_* = 4.1072e^−0.917*t*^	0.977	0.8
	anaerobic	C*_t_* = 3.3997e^−0.149*t*^	0.957	4.7
Cinnamon soil	aerobic	C*_t_* = 4.8526e^−0.278*t*^	0.983	2.5
	anaerobic	C*_t_* = 4.7710e^−0.060*t*^	0.973	11.6

**Table 6:** Transformation products of flonicamid in water and soil identified by UHPLC-Q-TOF/MS.

Compound Name	Formula	Observed *m*/*z* (Da)	Retention Time (min)	Mass Error (mDa)	Level
TFNG	C_9_H_7_F_3_N_2_O_3_	249.0474	3.52	−0.8	1
TFNA	C_7_H_4_F_3_NO_2_	192.0258	3.33	−0.9	1
TFNA-AM	C_7_H_5_F_3_N_2_O	191.0418	2.74	−0.9	1
TFNG-AM	C_9_H_8_F_3_N_3_O_2_	248.0634	2.74	−0.7	2b
TFNA-OH	C_7_H_4_F_3_NO_3_	208.0199	3.33	−1.7	2b
M210	C_9_H_4_F_2_N_2_O_2_	211.0305	2.74	−0.8	3

**Table 7:** Terminal residues of flonicamid and its metabolites in rice straw, rice husks and brown rice under different treatments.

Sample	Application Dose (g a.i./ha)	Harvest Interval(d)	Average Residue (mg/kg), n = 3			
			Flonicamid	TFNG	TFNA-AM	TFNA
Rice Straw	75	7	0.27	2.9	<0.01	0.040
		14	0.12	2.4	<0.01	0.055
		21	0.027	2.2	<0.01	0.060
	112.5	7	0.63	6.4	<0.01	0.10
		14	0.29	4.6	<0.01	0.20
		21	0.13	4.2	<0.01	0.31
Rice husk	75	7	1.7	0.40	0.018	0.070
		14	1.4	0.30	0.012	0.050
		21	1.2	0.63	0.037	0.060
	112.5	7	2.7	0.83	0.033	0.14
		14	2.1	0.80	0.025	0.11
		21	1.9	1.0	0.037	0.13
Brown rice	75	7	0.035	0.043	<0.01	<0.01
		14	0.024	0.050	<0.01	<0.01
		21	0.028	0.15	<0.01	0.019
	112.5	7	0.049	0.090	<0.01	<0.01
		14	0.072	0.12	<0.01	0.016
		21	0.062	0.19	<0.01	0.021

**Table 8:** Dietary risk assessment of flonicamid and metabolites in rice grain.

Population Group	%ADI/%											
	Flonicamid						Sum of Flonicamid, TFNG and TFNA					
	PHI 7d		PHI 14d		PHI 21d		PHI 7d		PHI 14d		PHI 21d	
	LD	HD	LD	HD	LD	HD	LD	HD	LD	HD	LD	HD
0–35 months female	0.501	0.702	0.344	1.031	0.401	0.888	1.145	1.990	1.088	2.921	2.749	3.808
0–35 months male	0.437	0.612	0.300	0.899	0.350	0.774	0.999	1.735	0.949	2.547	2.397	3.321
3–5 years female	0.457	0.640	0.313	0.940	0.365	0.809	1.044	1.814	0.992	2.663	2.506	3.472
3–5 years male	0.470	0.658	0.322	0.967	0.376	0.833	1.075	1.867	1.021	2.741	2.579	3.574
6–14 years female	0.350	0.490	0.240	0.720	0.280	0.620	0.800	1.390	0.760	2.040	1.920	2.660
6–14 years male	0.384	0.538	0.264	0.791	0.307	0.681	0.878	1.526	0.834	2.240	2.108	2.921
15–49 years female	0.226	0.316	0.155	0.464	0.180	0.400	0.516	0.896	0.490	1.315	1.237	1.714
15–49 years male	0.232	0.325	0.159	0.477	0.186	0.411	0.531	0.922	0.504	1.353	1.273	1.764
50–74 years female	0.350	0.490	0.240	0.720	0.280	0.620	0.800	1.390	0.760	2.040	1.920	2.660
50–74 years male	0.384	0.538	0.264	0.791	0.307	0.681	0.878	1.526	0.834	2.240	2.108	2.921
>75 years female	0.190	0.266	0.130	0.391	0.152	0.337	0.435	0.755	0.413	1.109	1.043	1.445
>75 years male	0.186	0.261	0.128	0.383	0.149	0.330	0.425	0.739	0.404	1.085	1.021	1.415

LD: low dose; HD: high dose.

## Data Availability

The original contributions presented in the study are included in the article and Supplementary Materials; further inquiries can be directed to the corresponding author.

## Supplementary Materials

The following supporting information can be downloaded at: https://www.mdpi.com/article/10.3390/foods15173104/s1, Figure S1. Matrix-matched calibration curves for flonicamid and its metabolites in different matrices. Figure S2. MS spectrum and proposed structures of the major fragments of M210. Table S1. Meteorological conditions and rice growth stages for each field application. Table S2. Linear ranges, regression equations, and correlation coefficients (*R*^2^) for flonicamid and its metabolites in matrix-matched solutions.
