# Supplementary material for: Residue Dissipation, Transformation Products, and Dietary Risk Assessment of Flonicamid in a Rice Paddy Ecosystem
Source: Foods. 2026 Sep 1;15(17):3104. doi: 10.3390/foods15173104 (PMC13565592; doi:10.3390/foods15173104)
Supplement: Supplementary file 1 [file foods-15-03104-s001.zip › foods-4495715-supplementary.pdf]

Supplementary Materials

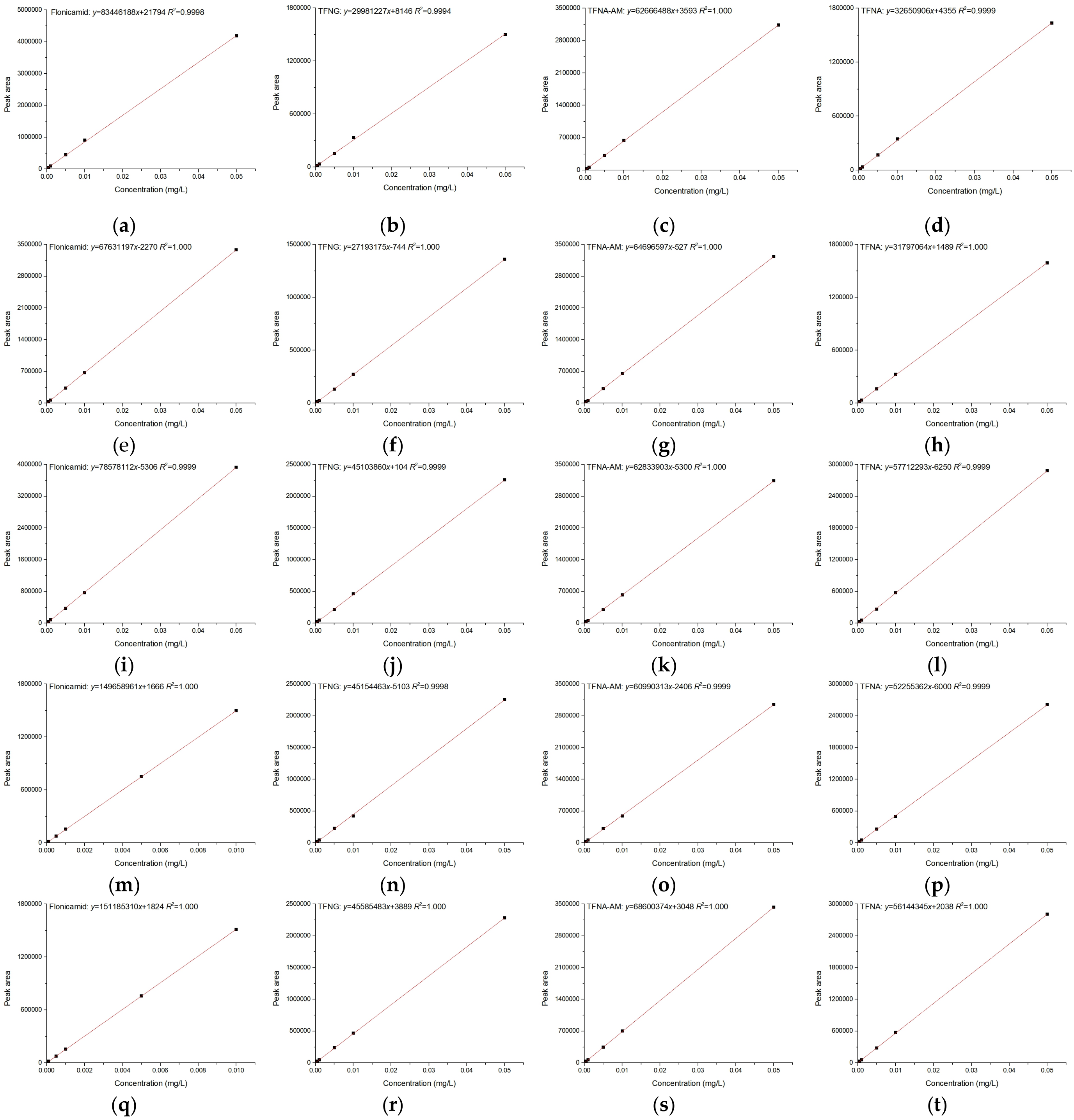

Figure S1. Matrix-matched calibration curves for flonicamid and its metabolites in different matrices. (a–d) Brown rice; (e–h) Rice husk; (i–l) Rice plant; (m–p) Soil; (q–t) water. Within each row, the panels from left to right correspond to flonicamid, N-(4-trifluoromethylnicotinoyl)glycine (TFNG), 4-(trifluoromethyl)nicotinamide (TFNA-AM), and 4-(trifluoromethyl)nicotinic acid (TFNA) , respectively.

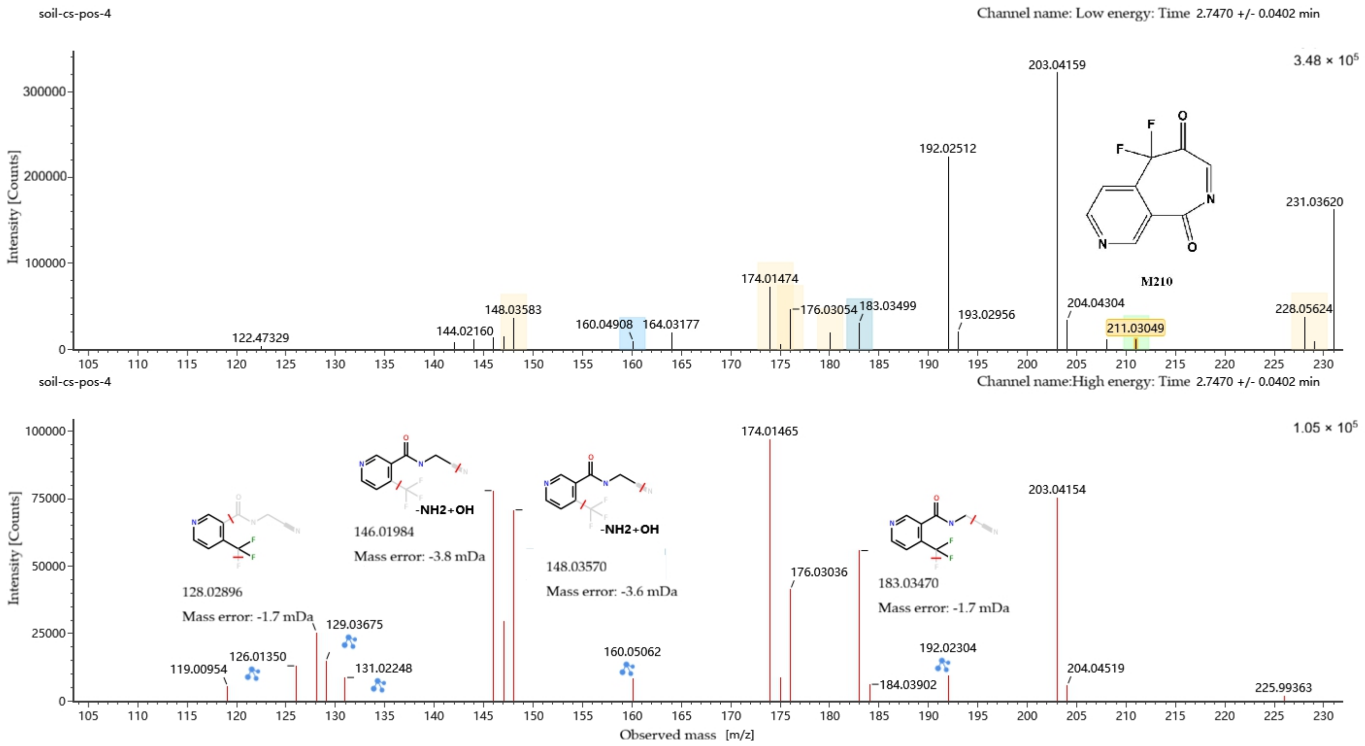

Figure S2. MS spectrum and proposed structures of the major fragments of M210

Table S1. Meteorological conditions and rice growth stages for each field application.

Period	Parameter	Dissipation (0 d)	Terminal residue (21 d)	Terminal residue (14 d)	Terminal residue (7 d)
At application	Application date	2024-05-06	2024-06-22	2024-06-29	2024-07-06
	BBCH stage	31	83	85	87
	Air temperature[1] (°C)	22.6	29.0	27.1	28.9
	Wind speed (m/s)	0.6	0.9	0.8	1.2
	Relative humidity (%)	76	85	81	68
	Weather condition	Partly cloudy	Cloudy	Cloudy	Partly cloudy
Before application	Last rainfall date[2]	2024-04-30	2024-06-19	2024-06-28	2024-06-30
	Days from application (d)	-6	-3	-1	-6
	Rainfall amount (mm)	2.99	1.92	9.86	2.36
After application	First rainfall date	2024-05-30	2024-06-24	2024-06-30	2024-07-13
	Days from application (d)	24	2	1	7
	Rainfall amount (mm)	4.45	1.92	2.36	33.84

[1] On-site measurements of air temperature, wind speed, and relative humidity were performed with a handheld anemometer and a thermohygrometer during each application.

[2] Rainfall data were obtained from Tianqi24 [38] for the meteorological station nearest to the experimental site.

Table S2. Linear ranges, regression equations, and correlation coefficients (R^2) for flonicamid and its metabolites in matrix-matched solutions.

Matrix	Compound	Linear range (mg/L)	Regression equation	Correlation coefficient (R^2)
Brown rice	Flonicamid	0.0001–0.05	y = 83446188x + 21794	0.9998
	TFNG	0.0001–0.05	y = 29981227x + 8146	0.9994
	TFNA-AM	0.0001–0.05	y = 62666488x + 3953	1.000
	TFNA	0.0001–0.05	y = 32650906x + 4355	0.9999
Rice husk	Flonicamid	0.0001–0.05	y = 67631197x - 2270	1.000
	TFNG	0.0001–0.05	y = 27193175x - 744	1.000
	TFNA-AM	0.0001–0.05	y = 64696597x - 527	1.000
	TFNA	0.0001–0.05	y = 31797064x + 1489	1.000
Rice plant	Flonicamid	0.0001–0.05	y = 78578112x - 5306	0.9999
	TFNG	0.0001–0.05	y = 45103860x + 104	0.9999
	TFNA-AM	0.0001–0.05	y = 62833903x - 5300	1.000
	TFNA	0.0001–0.05	y = 57712293x - 6250	0.9999
Soil	Flonicamid	0.0001–0.01	y = 149658961x + 1666	1.000
	TFNG	0.0001–0.05	y = 45154463x - 5103	0.9998
	TFNA-AM	0.0001–0.05	y = 60990313x - 2406	0.9999
	TFNA	0.0001–0.05	y = 52255362x - 6000	0.9999
water	Flonicamid	0.0001–0.01	y = 151185310x + 1824	1.000
	TFNG	0.0001–0.05	y = 45585483x + 3889	1.000
	TFNA-AM	0.0001–0.05	y = 68600374x + 3048	1.000
	TFNA	0.0001–0.05	y = 56144345x + 2038	1.000
